# Recent Advances on Conducting Polymers Based Nanogenerators for Energy Harvesting

**DOI:** 10.3390/mi12111308

**Published:** 2021-10-25

**Authors:** Weichi Zhang, Liwen You, Xiao Meng, Bozhi Wang, Dabin Lin

**Affiliations:** 1 Mechanical Engineering, The University of Sydney, Sydney, NSW 2006, Australia; 2 School of Materials Science and Engineering, East China University of Science and Technology, Shanghai 201424, China; 15190623806@163.com; 3 Shaanxi Province Key Laboratory of Thin Films Technology and Optical Test, School of Optoelectronic Engineering, Xi’an Technological University, Xi’an 710032, China; xiaomengxatu@126.com (X.M.); 19591526681@163.com (B.W.)

**Keywords:** nanocomposites, conducting polymer, energy harvesting, PENG, TENG

## Abstract

With the rapid growth of numerous portable electronics, it is critical to develop high-performance, lightweight, and environmentally sustainable energy generation and power supply systems. The flexible nanogenerators, including piezoelectric nanogenerators (PENG) and triboelectric nanogenerators (TENG), are currently viable candidates for combination with personal devices and wireless sensors to achieve sustained energy for long-term working circumstances due to their great mechanical qualities, superior environmental adaptability, and outstanding energy-harvesting performance. Conductive materials for electrode as the critical component in nanogenerators, have been intensively investigated to optimize their performance and avoid high-cost and time-consuming manufacture processing. Recently, because of their low cost, large-scale production, simple synthesis procedures, and controlled electrical conductivity, conducting polymers (CPs) have been utilized in a wide range of scientific domains. CPs have also become increasingly significant in nanogenerators. In this review, we summarize the recent advances on CP-based PENG and TENG for biomechanical energy harvesting. A thorough overview of recent advancements and development of CP-based nanogenerators with various configurations are presented and prospects of scientific and technological challenges from performance to potential applications are discussed.

## 1. Introduction

A variety of highly flexible and high-performance electronic devices have been developed for next-generation applications in different fields, including health monitoring, smart communication, flexible displays, energy storage, green electronics, and artificial intelligence systems [1,2,3]. The increasing demand for the integration of electronic technology into flexible and wearable devices is driven by the need for continuous, long-term, and individual monitoring at any location and at any time, which is becoming increasingly important. Many different types of wearable and flexible electronics have been proposed to detect physical activity and bio-signals from the human body, including biomechanical signals (bending, pressure, motion, tactile, vibration, heartbeat, breath), temperature, humidity, and electrograms (electromyography, electrocardiography, electro-oculography, and electro-encephalography) [4]. Our bodies consist of a variety of energies, such as chemical, thermal, and mechanical energy, the latter of which is the most plentiful. In recent decades, wearable energy-harvesting systems and generators have been increasingly significant in recent decades when it comes to self-powered electronic sensors and devices. The research is primarily concerned with the extraction of the greatest amount of useable energy and the conversion of this energy into electric energy. Traditional energy harvesters, on the other hand, continue to encounter difficulties in properly capturing motion, in order to boost the power production while maintaining the system′s stability. However, their improvements must still face numerous hurdles, including how to minimize weight and size, as well as the restricted power supply. As a result, downsizing of flexible, intelligent, and self-powered wearable devices has emerged as a critical research area. To utilize this mechanical energy and address the previously described obstacles, a nanogenerator (NG) that can convert mechanical energy into electric energy was designed in 2006; it has made amazing progress in the last 15 years. There are two types of devices that transform small quantities of mechanical energy into electrical energy via the piezoelectric or triboelectric effects, known as piezoelectric nanogenerators (PENGs) and triboelectric nanogenerators (TENGs) [5].

Body motions that are observed and used can be divided into two categories: limb motion (i.e., walking, running, jogging, bending, stretching) and subtle motion (i.e., skin tension, heart beats, breathing, blood pressure, phonation) [6]. Human physical vital signs generate strain and/or pressure changes are collected and converted to electrical signals and energy by nanogenerators, which are significant markers for human energy harvesting. Generally speaking, it may be classified into two categories: (i) energy with mild strain variation, such as heartbeat and blood pressure; (ii) energy with large strain variation, such as variable moving states, finger bending, and facial expression. The major criteria and challenges for high-performance flexible nanogenerators are to: (i) detect output signals sensitively and in a timely manner, (ii) have better sensing capabilities with resilience and recyclability, and (iii) limit potential pollution as green electronics.

Traditional wearable electronics are generally made of silicon and carbon-based materials, which have poor biological compatibility and a hard substrate for skin adhesion. Among them, because of their high electrical conductivity, simplicity of synthesis, and innate flexibility, conducting polymers (CPs) have been considered the most suitable materials for flexible electronics [7]. Polymers are usually considered as electrical insulators before the discover of conducting polymers; yet, these organic polymers exhibit distinctive electrical and optical properties, which are comparable to widely used inorganic semiconductors. The electrical and optical properties of CPs are attributed to the conjugated carbon chains, which are composed of alternating single bonds and double bonds and are determined by highly delocalized, oriented, and electron-dense p bonds. Typical CPs include polyacetylene (PA), polyaniline (PANI), polypyrrole (PPy), poly(3,4-ethylenedioxythiophene):poly(styrenesulfonate) (PEDOT:PSS), poly- thiophene (PTH), poly(para-phenylene) (PPP), poly(phenylenevinylene) (PPV), and polyfuran (PF). The different methods of CPs synthesis and fabrication have been well discussed based on our research experience [8,9,10]. Conducting polymers with high flexibility and stretchability, as well as mechanical and electrical responsiveness, have contributed to the recent acceleration of the study on CPs-based in flexible and wearable electronics. Achieving electrical control over a film′s volume and conductivity has been achieved according to the tunable electrical conductivities by doping and de-doping of the polymer. Chemical polymerization, electrochemical polymerization, and photo-induced polymerization are all methods for producing conducting polymers with a variety of nanostructures by controlling the nucleation and growth processes of polymerization. Furthermore, hybridization of CPs with other nanomaterials such as metal, metal oxides, chalcogenides, carbonaceous materials, and metallic oxide resulted in promising functional nanocomposites with improved harvesting performance.

**Figure 1:**
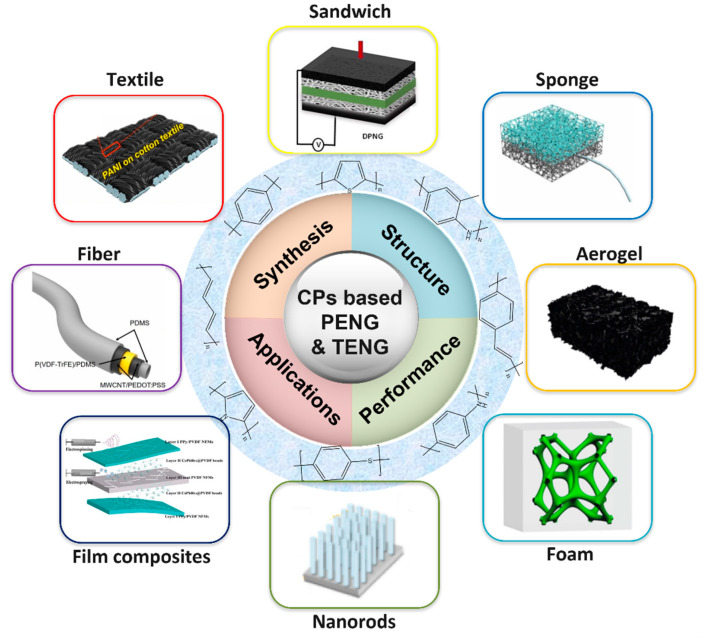
Schematic of overview of conducting polymers assisted nanogenerators with different structures for energy harvesting, including sandwich [26], sponge [27], aerogel [28], foam [29], film composites [30], filers [31], nanorods [32], and textile [33].

Until now, there are several published review papers about conductive polymer nanocomposites and flexible electronics for sensing and biomedical applications. Mokhtar et al. gave a mini review on conducting polymers in wearable devices. Liu et al. reviewed the fabrication and structures of conductive polymer composites for flexible strain sensors [11]. In addition, there are also some review articles about flexible conductive polymer composites for biomechanical sensors, which not only focus on conducting polymers, but also include other conducting (metal, carbonaceous, metallic oxide) fillers [12,13,14,15]. Some review articles summarized conducting polymers for sensing [7,16], energy storage [17], environmental protection [18], and electromagnetic interference shielding [19], but did not focus on the biomechanical energy harvesters [20,21]. Furthermore, research also provided summaries for some conducting polymers, such as PEDOT [22,23], PANI [24] and PPy [25]. However, at present, the conducting polymers nanogenerators with various structures for wearable energy harvesting have not been systematically summarized. Different to the previous study, this review aims to summarize the recent advances on CP-based PENG and TENG for biomechanical energy harvesting. As shown in Figure 1, CP-based nanogenerators with various structures and related performances are presented and prospects of challenges to potential applications are discussed.

## 2. Conducting Polymer Based Piezoelectric Nanogenerators

**Figure 2:**
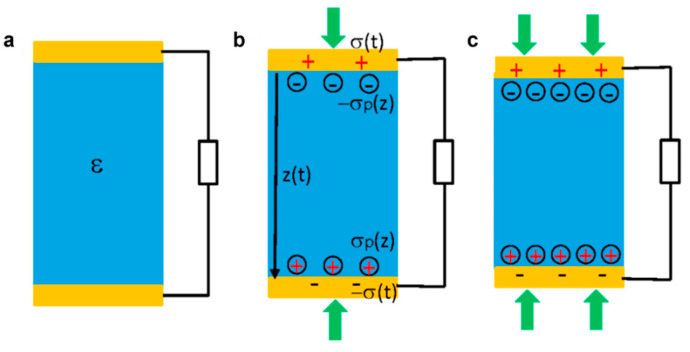
The working principle of a piezoelectric nanogenerator (PENG): (**a**) An insulator piezoelectric material is covered by top and bottom electrodes on its two surfaces. (**b**) A vertical mechanical deformation results in the generation of piezoelectric polarization charges at the two ends of the material. (**c**) An increase of the applied force results in higher polarization charge density [34].

The piezoelectric nanogenerator is a sustainable alternative that converts mechanical energy from its surroundings into electrical power. Different from the working principle of TENG, by segregating negative and positive charges between piezoelectric materials under an externally applied stress, PENG can generate an electron flow to an external load [5,34]. In Figure 2, top and bottom electrodes cover an insulator piezoelectric material. Piezoelectric polarization charges are created by vertical mechanical deformation. The polarization charge density increases with applied force. The external load moving electrons between electrodes balances the electrostatic potential created by the polarization charges. This is how mechanical energy becomes electrical energy. When the strain generated by applied force is taken into account, the density of piezoelectric polarization charges on the surface is σ_p_(z), and the density of free electrons in the electrode is σ(x) [35]. Furthermore, PENG collecting energy is more favorable since it can be widely used to diverse sites while being less affected by environmental circumstances such as humidity and temperature. Researchers are studying piezoelectric materials such as crystals, synthetic ceramics, and polymers extensively, developing these materials at the nanoscale to improve piezoelectricity. PENG is classified into two types: those that combine piezoelectric nanoparticles with non-piezoelectric matrices, and those that use piezoelectric polymers as both functional materials and matrices.

Self-powered piezoelectric harvesters have been built using a variety of classic piezoelectric materials and ceramics. Tremendous efforts have been made to improve the dielectric constant and mechanical properties of PENG by including nanofillers such as synthetic crystals/ceramics and 2D materials into a piezoelectric or other polymer matrix, which has been shown to be effective. It is true that the manufacture of this device involves the use of nanomaterials such as nanowires, microparticles, and nanoflakes [36]. Similar to the dielectric energy storage devices [37,38,39,40,41], the synthetic crystal/ceramic components, such as barium titanate (BaTiO_3_) [42], lead zirconate titanate (PZT) [43], Zinc sulphide (ZnS) [44], and ZnO [45,46] were proposed in many studies in the literature to improve the energy conversion efficiency and output performance [47]. The high surface-to-volume ratio, which is particularly prevalent in nanomaterial composites, has the tendency to produce specialized nanogenerator characteristics. However, despite their high dielectric constant, piezoelectric ceramics are generally brittle, have a low dielectric breakdown, and are difficult to manufacture. Increasing the sturdiness of mechanical energy-harvesting PENG so that it can withstand severe stress and strain is crucial.

Due to of its high piezoelectric coefficient, high breakdown field, low-temperature manufacturing, low cost, and mechanical flexibility, poly(vinylidene fluoride) (PVDF) and copolymers have obtained much attention as functional films for energy harvesting from ambient mechanical vibrations [48,49,50,51,52,53]. Only the β and γ crystalline phases contribute to piezoelectricity due to the arrangement of high electronegativity bonds between C-F atoms, producing in a dielectric moment that generates an electric field [54,55]. When a mechanical action is applied to the β-phase macromolecule, various charge concentrations occur on different sides of the chain. In addition, other PVDF based co-polymers, Poly(vinylidene Fluoride-Trifluoroethylene) [P(VDF-TrFE)], Poly(vinylidene fluoride-hexafluoropropylene) [P(VDF-HFP)], Poly(vinylidene fluoride-chlorotrifluoroethylene) [P(VDF-CTFE)], and P(VDF-TrFE-CFE) terpolymers, have been used for energy storage and harvesting devices [56,57,58].

Recently, conducting polymers have two main functions in PENG: one is to act as a filler to increase piezoelectric performance, and the other is to act as an electrode to improve electrical conductivity. In this section, the CPs-assisted PENG will be summarized and discussed to show their role in different configurations, including sandwich structure, porous structure, and textile structure. The key parameters of selected PENG are listed in Table 1.

### 2.1. Sandwich Structured PENG

In contrast to TENG, the sandwich structure of electrode/piezoelectrics/electrode is the primary configuration in PENG. The PEH performance enhancement in terms of electrical power is a critical issue in their optimization. Many research groups have focused on the modification of active materials and electrodes. Because it is made from bulk conversion with a small thickness and a surface area of only a few square centimeters, the sandwich with functional thin film is utilized. One of the most common methods for increasing the functional area, while keeping the device compact, is to improve the film′s specific surface area per unit volume through nanostructuring. The magnitude and sensitivity of the piezoelectric signal are expected to improve as a result. Many nanostructures regularly used for this purpose, such as nanorods, nanowires, nanobelts, nanotubes, and so on, are vertically arranged [46,70].

**Figure 3:**
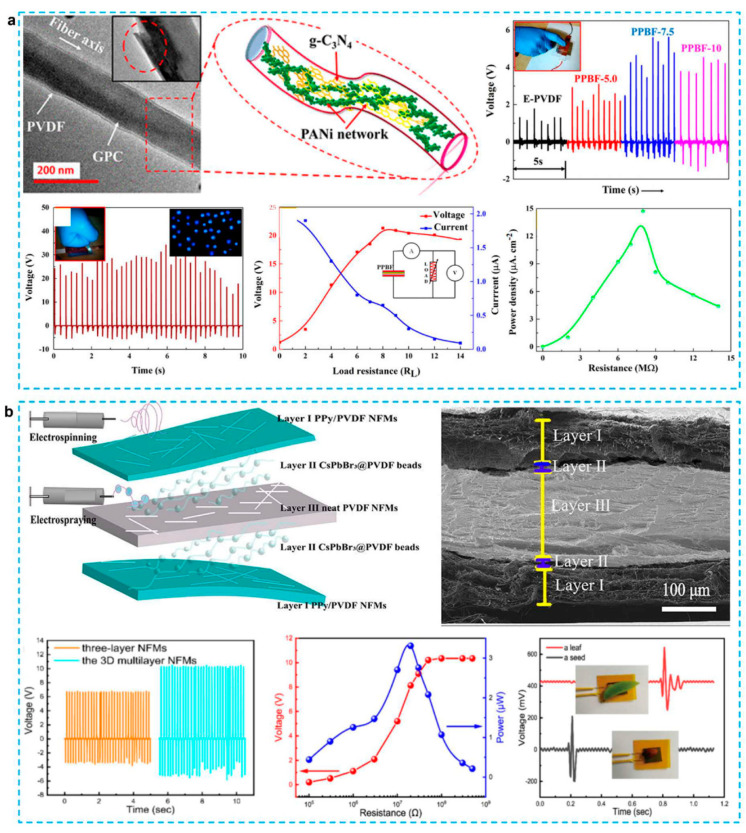
(**a**) Polyaniline-graphitic carbon Nitride nanosheet composite materials with embedded PVDF nanofibers for piezoelectric energy conversion, including TEM morphology, Piezoelectric voltage output for different samples [63]; (**b**) Electrospun/electrosprayed PVDF-based nanofiber and bead multilayer assembly with increased piezoelectricity and high sensitivity, including schematic of the 3D multilayer, cross-section SEM image and generated open-circuit output voltage and short-circuit current [30].

To begin, the CPs can be utilized to improve the performance of nanostructured piezoelectric materials. The use of electrospinning to produce PVDF nanofibers has been shown to improve the piezoelectricity of PVDF. The piezoelectric behavior of PVDF is improved by poling and mechanical stretching of nanofibers at the same time. Electrospun nanofibers have a high aspect ratio, a large surface area, high porosity, and a low weight. Recently, PVDF/PANI nanofibers for energy harvesting were investigated [63,71,72]. The use of conductive fillers such as metal, CNT, graphene, and PANI aids in the formation of an electrically conductive network [73,74,75,76,77,78,79], which improves the piezoelectric conversion efficiency. However, CNT and graphene are very easy to substantial aggregation within the PVDF nanofibers. The use of PANI has no effect on the induction of β-phase in PVDF. A series of work on flexible electrospun PVDF/PANI nanofiber based film for piezoelectric nanogenerator have been reported [30,63,68,80,81]. The additional fillers, such as reduced graphene oxide [81], halloysite nanotube [68], graphitic carbon nitride nanosheets [63], and perovskite nanoparticles [30], also have been introduced to the composites to help in the formation of electrically conducting network. For example, Figure 3a represents that PANI nanorods and g-C_3_N_4_ were integrated into PVDF nanofibers to improve the piezoelectric performance [63]. When compared to pristine PVDF nanofibers, which showed a 1300% increase in voltage and current output. With high stability and reproducibility (> 50 000 cycles), the nanogenerator produced a voltage output of 30 V and a current output of 3.7 A. Li et al. made a 3D multilayer PENG combining PPy electrodes, electrospun PVDF, and electrosprayed CsPbBr3@PVDF beads, in which two layers of CsPbBr3@PVDF beads were placed between the top/bottom PPy electrodes and PVDF nanofibers layer, as shown in Figure 3b [30]. The distribution and number of perovskite nanoparticles in PVDF are crucial for achieving outstanding output performance of composite-based PENG. The 3D multilayer PENG showed a high open-circuit voltage of 10.3 V and a short-circuit current density of 1.29 A/cm^2^, and could sense a low pressure as small as 7.4 Pa.

Besides the modification of the piezoelectric functional materials by CPs, in the second approach, the CPs can be utilized to improve the performance of the electrode in the sandwich (electrode/piezoelectrics/electrode) structure, which is another critical aspect of PENG [82]. Metal nanowires (i.e., Ag nanowires) electrode is the promising candidate because of its excellent mechanical performance and good conductivity [83]. However, due to surface roughness and poor adherence to wire joints and substrates, electrode films made of metal nanowires are not appropriate for large-scale devices. The doped indium oxide (ITO) exhibited high electrical conductivity and optical transmittance, but also has limited application due to the scarce and complicated preparation process. To solve these issues, the hybrid CPs with other conducting nanomaterials have been intensively proposed to avoid poor rigidity and bending resistance of traditional electrodes and improve the flexibility and conductivity [42,62,66,81,84,85]. These are promising methods for overcoming the differences in the local effective electric field in the nanocomposite, which results from the differences between the fillers and polymer phases in the electric field [65].

**Figure 4:**
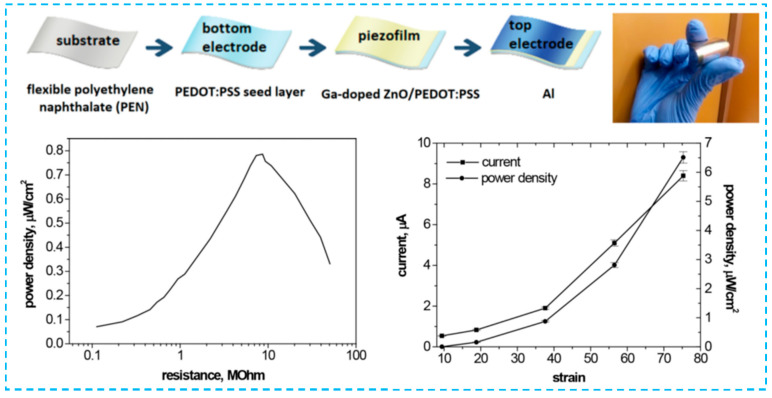
Polymeric seed layer as a simple approach for nanostructuring of Ga-doped ZnO films for flexible piezoelectric energy harvesting [45].

In the metal/CPs nanocomposites, the Ag nanowires and CPs can be used not only as and bottom electrodes separately [61], but also integrated with CPs for both electrodes [86]. A high diffusive transmittance of 84% at 550 nm and a small sheet resistance of 17 Ω/sq were achieved when PEDOT:PSS mixed with Ag nanowires in electrodes for PVDF-HFP piezoelectric nanogenerators. PEDOT:PSS-PVP nanofiber membrane integrated with CNT as the electrodes [87]. The PENG can also work on both of body motion and cold/hot airflow thanks to its flexible non-woven structure. rGO and conjugated conductive polymers have been employed as electrodes or nano-additives in nanogenerators made of carbon/CPs composites for electrodes. Unsal et al. found that PANI with rGO resulted in a synergistic effect and produced an enhanced voltage compared to other composites [81]. The CPs can also be used as a bottom electrode and as an amorphous seed sublayer for nanostructured piezoelectric nanostructures. Ga-doped ZnO piezoelectric film was grown on amorphous seed layer PEDOT:PSS produced by spray deposition on a flexible substrate using RF sputtering with MEMS technology [45]. As illustrated in Figure 4, for structures having a seed polymer layer, an increase in piezoelectric voltage is obtained. The work′s importance stems from the discovery of fresh information regarding unique nanostructured piezoelectric films on polymers with dual functions (seed layer and electrode), as well as the definition of their operational parameters and prospective uses.

**Figure 5:**
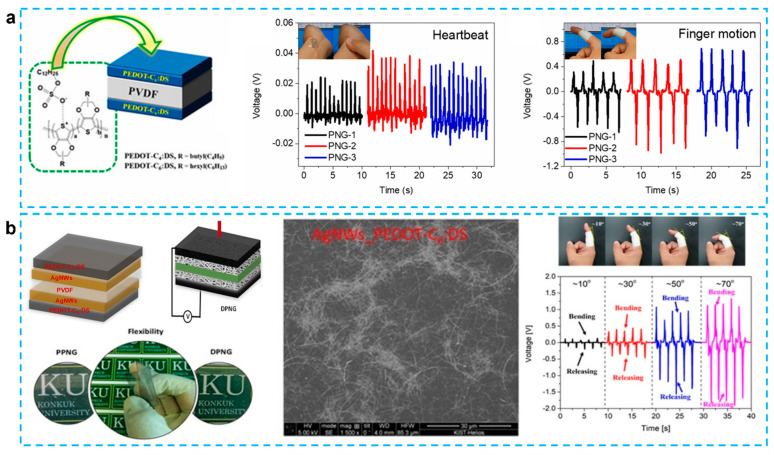
(**a**) Schematic illustration of sandwich structure with PEDOT-Cx:DS. The generated output voltage performance from the PNGs used for heartbeat monitoring system attached to wrist and human motion monitoring system attached to index finger [59]. (**b**) Schematics of DPNG, Photographs of transparency of AgNWs film, Flexible PVDF based hybrid electrode PNG and transparency of hybrid electrode, cross-section SEM images of DPNG, Performance of flexible DPNG under index finger movement [26].

Besides mixing CPs with other conducting electrodes, modification of PEDOT is also an important strategy to improve the conductivity and enhance the performance. As shown in Figure 5a, a nanofiber-type 1D poly(2-hexyl-2,3-dihydrothieno[3,4-b][1, 4]dioxine:dodecyl sulfate (PEDOT-C6:DS), which exhibited conductivity of 50 S/cm (42 times of PEDOT:PSS), has been proposed to improve the performance of PENG [59]. With a hydrophobic surface and a homogeneous conducting network on PVDF, the polymer material possesses good solubility and dispersion in organic solvents, as well as good electrical conductivity. In comparison to the PEDOT:PSS-CNT electrode, the maximum voltages/currents of this device utilizing these materials as electrodes exhibited 63.0 nW at 9 M, which was increased 54%. Further work illustrated in Figure 5b using the hybrid electrode consisting of AgNWs and PEDOT-C6:DS also obtained good results [26]. In another study, the acrylic monomers 4-hydroxybutyl acrylate (HBA) and 2-carboxyethyl acrylate (CEA) were co-polymerized with styrene sulfonate to synthesis two different forms of anionic polyelectrolytes, P(SS-co-HBA) and P(SS-co-CEA), respectively [67]. Compared with PENG with PEDOT:PSS, the maximum output power improved by 27.4% and 7.8%. The reason for this is that in both composites, crosslinking came as a result of an increase in annealing temperature following film casting, which increased electrical conductivity and hydrophobicity when compared to PEDOT: PSS.

### 2.2. Textile Structured PENG

Textile-based energy harvesters, which directly used piezoelectric fibers or used substrates such as cotton, polyester, and nylon, have such a great potentiality for wearable electronic devices because of their softness, excellent flexibility, great durability, and capability to be combined with clothing. Fiber-based electronics, which may be woven into textiles to give improved comfort, robustness, and integrated multi-functionalities, are predicted to become increasingly essential in the near future as emerging generations of wearable electronics are likely to be directly worn.

**Figure 6:**
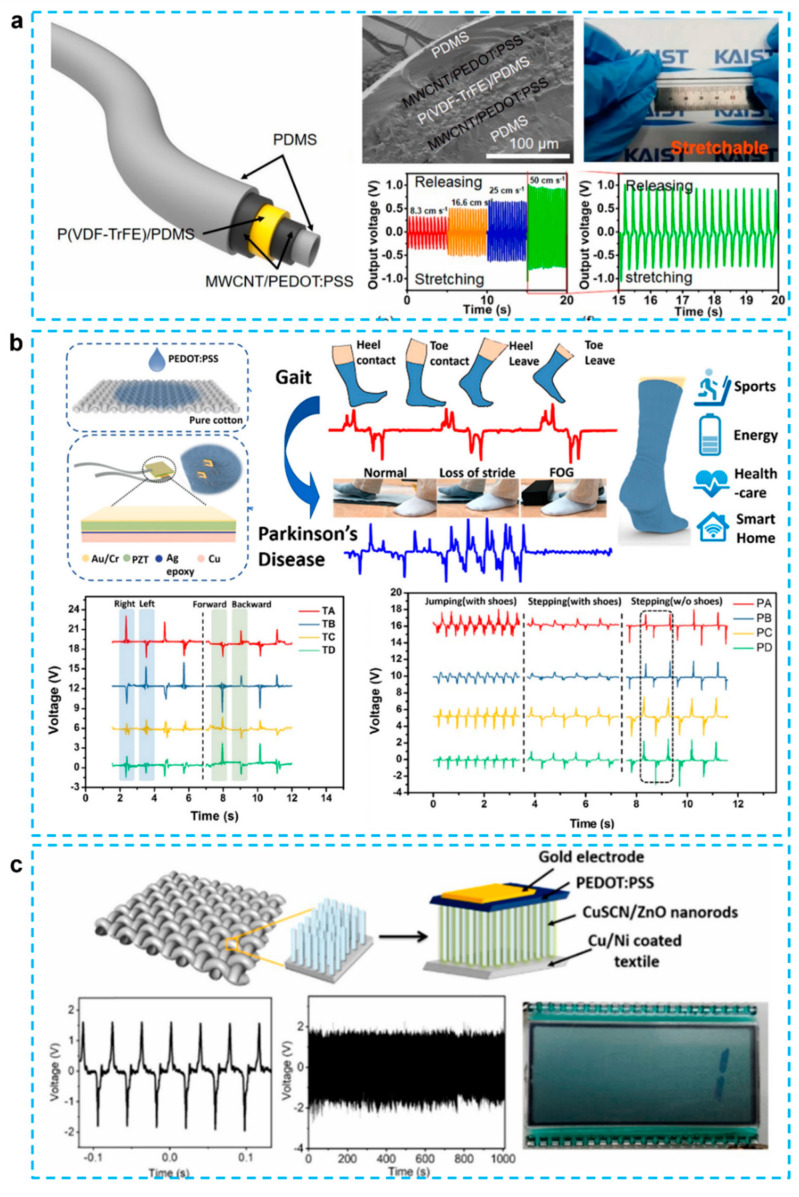
(**a**) Intrinsically stretchable multi-functional fiber with energy harvesting and strain sensing capability [31]. (**b**) Self-powered and self-functional cotton sock using piezoelectric and triboelectric hybrid mechanism for healthcare and sports monitoring [43]. (**c**) P-N junction-based ZnO wearable textile nanogenerator for biomechanical energy harvesting [32].

Fiber-based electronics are gaining popularity because they can be woven into a variety of 3D designs using well-established textile production processes. Various piezoelectric materials have been employed to create fiber-based energy-harvesting devices in this regard [88,89,90]. However, these devices are not flexible, restricting their ability to completely harvest the various mechanical energy sources. Ryu et al. demonstrate a multi-functional hollow fiber that is innately stretchy and able to collect mechanical energy and detect strain changes [31]. As shown in Figure 6a, the stretchy P(VDF-TrFE) in an elastomer matrix sandwiched between MWCNT and PEDOT:PSS is used for energy harvesting. The inherent stretchability of these layers allows for efficient energy harvesting from a wide range of mechanical stimuli. Under stretching and normal pressure, the researchers exhibited voltage and current generation, with output voltage of 1.2 V and current of 10 nA. As evidenced by the harvesting pressure generated by liquid flow, the hollow architecture of our fiber allows energy to be harvested from radially outward internal pressure.

Very recently, CPs-assisted PENG in the smart textile have become a very interested research topic. Zhu et al. have created a self-powered and self-functional sock that can perform a variety of tasks, such as energy harvesting and sensing different physiological signals by hybrid integrating PEDOT:PSS-coated fabric PENG, as depicted in Figure 6b [43]. A PEDOT:PSS-coated sock with mild jumping at 2 Hz and load resistance of 59.7 M produces an output power of 1.71 mW, and the measured piezoelectric power density for PZT chips is 128 W/cm^2^. This sock can also perform a variety of functions, including energy harvesting, motion tracking, walking recognition, gait sensing, and sweat sensing. Researchers observed that using CP-based top electrodes to produce p-n junction-based nanogenerators could offer various advantages for a textile-based nanogenerator because of the non-planar surface involved and the great flexibility of the textile substrate. Figure 6c presented a p-n junction PEDOT:PSS/CuSCN-coated ZnO textile nanogenerator embedded in textiles, which combines the benefits of ZnO piezoelectricity with textile flexibility [32]. It has been revealed that average length of ZnO nanorods increases the output voltage and power density of nanogenerators. As the shaking frequency increases from 19 Hz to 26 Hz, the device with optimized ZnO nanorod length produces an increasing output voltage ranging from 0.2 V to 1.81 V, which may activate an LCD screen display. These findings will be especially useful in the creation of future self-powered flexible electronics.

## 3. Conducting Polymer Based Triboelectric Nanogenerators

As discussed in the introduction, in contrast to conventional power supply, which has a short service life and requires extensive maintenance, triboelectric nanogenerators (TENGs) have been developed fast and been considered as a multipurpose platform for constructing self-powered sensor systems and wearable electronics, as well as a promising energy conversion technology. With the advantages of being low-cost, lightweight, high-efficiency, and having a wide range of materials available, TENG have been used in self-charging power systems, active sensors, and sustain-able energy sources, among other applications.

**Figure 7:**
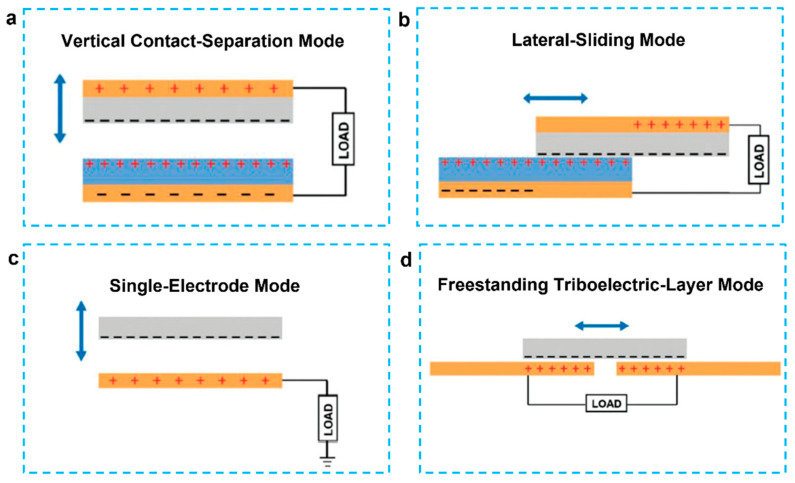
(**a**) Four working modes of TENGs. (**a**) Vertical contact-separation mode. (**b**) Lateral-sliding mode. (**c**) Single-electrode mode. (**d**) Freestanding triboelectric-layer mode [91].

Since its first report in 2012, four different operation modes of the TENG have been proposed, as shown in Figure 7, based on the direction of polarization change and electrode configuration: vertical contact-separation (CS) mode, lateral-sliding (LS) mode, single-electrode (SE) mode, and freestanding triboelectric-layer (FT) mode [91,92]. When relative motion occurs during contact-electrification, oppositely polarized triboelectric charges can develop on the material′s surfaces. TENGs are a form of capacitive variable electric field source based on Maxwell′s displacement current, and their output power is proportional to triboelectric charge density squared. Increasing the quantity of produced triboelectric charges is the key to boosting TENG performance [93,94].

To deposit or coat on conductive electrodes, triboelectric materials with either positive or negative polarity are utilized. Various conductive materials, such as metals and carbon-based compounds, have been used as electrodes thus far [95]. Metal-based materials, on the other hand, are easily oxidized or corroded in a hostile environment during the chemical or electrochemical preparation of the nano-structured metals, which can have a significant impact on the long-term durability of the resulting TENGs [96]. Polymeric materials, which are shape adaptable, are another option. Smart electronics and biosensors have provided an inexhaustible impetus for the evolution of the conducting polymer-based ecosystem. Since Wang et al. first demonstrated that PPy can be used to make electrodes and triboelectric layers [97,98], because of their large electrical conductivity and specific capacitance, remarkable chemical and thermal stability, simplicity of synthesis, and low cost, CPs have been recognized as one of the most promising electrode materials. Furthermore, CPs demonstrated robust functioning in a normal ambient environment and were biocompatible. Several 3D structured CPs-based TENG have been reported, including knitting power textile, cotton textile, 3D printing patterns, and sandwich layers. In this section, the role and structure of CPs for improving the performance of TENG is discussed, including multilayer structures and various 3D porous structures. The key parameters of selected TENG are listed in Table 2.

### 3.1. Nanostructured Films Based TENGs

TENGs based on CPs have recently been intensively explored and used for a variety of energy conversion and storage devices. Composite materials with a good trade-off between mechanical compliance and triboelectric characteristics can be developed by modifying the chemical properties of conducting polymers and introducing additives [103,109]. Their contact resistance in the microstructure could be used to quantify external pressures. A CPs-based composite can be changed by applying pressure on it [98]. Besides the conductive coating layers, numerous 3D structures have been proposed to not only allow a choice of production processes for self-charging power devices but also to adapt to mechanical shape changing. Regarding the exceptionally hard working conditions of wearable devices, such as bending, pressing, stretching, and twisting, a fully shape-adaptive, flexible, and stretchable self-charging power 3D structure is the most desirable solution due to the excessive stretching, bending, and twisting. As the triboelectric effect creates the electric potential difference, an efficient method of improving power output was established by inserting micro- and nano-scaling topographies on the material surface. Many previous investigations used a micropatterned pyramid array, a nanoporous/nanowire structure, and chemical functional groups to alter the tribo-material in order to achieve a high charge density on the surface [110,111,112].

**Figure 8:**
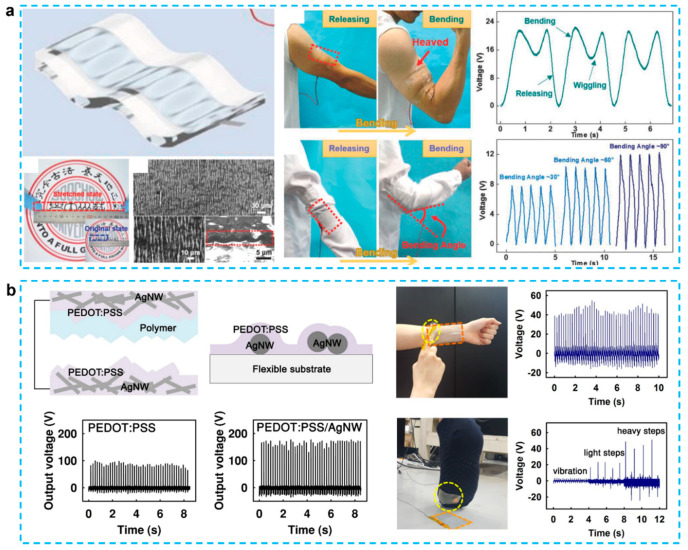
(**a**) A wrinkled PEDOT:PSS film-based stretchable and transparent triboelectric nanogenerator for wearable energy harvesters and active motion sensors [107]. (**b**) Transparent and flexible high power triboelectric nanogenerator with metallic nanowire-embedded tribonegative conducting polymer [101].

Usually, in the CPs-based TENG, the nanostructured CPs grown on the substrate act as both frictional layer and conducting electrode [97,100]. Triboelectricity needed a huge difference in triboelectric polarity between a contact and an electrode. It is critical to design the integration of triboelectric layer and electrode as a uniform structure for stretchable or elastic TENGs. A single-electrode mode TENG as a consequence of the coupling effects of triboelectrification and electrostatic induction can explain the functioning process [107]. As shown in Figure 8a, to create wearable energy harvesters, Wen et al. presented a wrinkled PEDOT:PSS film-based triboelectric nanogenerator. The PEDOT:PSS electrode has a conductivity of 0.14 k and a transparency of 90% at 100% strain. When operated in single-electrode mode at 2.5 Hz, the TENG with the area of (6 × 3 cm^2^) produces an open-circuit voltage of 180 V, a short-circuit current of 22.6 A, and an average power density of 4.06 mW/m^2^. In around 3.5 min, it can charge the capacitor to 2 V and power an electronic watch when worn on the wrist. The TENG could also measure the bending angle of the elbow and joint by measuring the peak voltage and counting the peak number as a human motion sensor. To attain good output performance, PEDOT:PSS with a moderate triboelectric negativity must be connected with a charge-donating counter material [101]. As illustrated in Figure 8b, in the PEDOT:PSS with Ag NW embedded film, the output voltage is around 170 V, which is more than twice of the value without the AgNW layer. The reason can be summarized as: (i) a set of contact materials with a large difference in the opposing triboelectric polarity was chosen to boost the TENG′s output performance; (ii) AgNWs were added to PEDOT:PSS to improve the contact electrode′s conductivity and surface roughness; and (iii) the PUA counter contact material was micro-structured via soft lithography imprinting to increase the effective contact area.

### 3.2. Sponges/Foam/Aerogel Structured TENG

To achieve excellent TENG performance, various CPs-based 3D nanostructured highly porous composites, such as sponge, foam, and aerogels, have recently been proposed. The development of highly porous composites and nanostructured CPs over the last few decades has resulted in a wide range of applications that benefit from their ease of fabrication, scalability, highly porous structures, super-lightweight nature, moderate mechanical properties, and large surface areas [19]. They have been investigated as new functional composites in water remediation [113], liquid transport [114], electronic devices [115], supercapacitors [116], and other applications [117]. Very recently, researchers found that the incorporation of porous structure into TENGs can create a new way for the production of TENGs. There are mainly three types: the first is interconnected structured sponges/foams (i.e., graphene and metal foam-based sponges); the second is and elastic polymers based sponges (i.e., PDMS or PU sponges); the third is the aerogels obtained from nature plants (i.e., Cellulose nanofibrils based aerogels).

A conductive elastic sponge was used to create an elastic PU-based TENG, which has potential applications in a variety of mechanical energy-harvesting applications. The conductive elastic sponge is created by drying a dilute chemical polymerization of aniline on an elastic sponge. The outstanding performance can be attributed to two aspects: (i) the sponge′s ability to harvest irregular and random mechanical energy ubiquitous in the surrounding environment due to its soft and elastic characteristics, and (ii) the sponge′s capacity to efficiently transform external inputs into electrical signals as a result of the presence of nanostructured CPs on the sponge′s surface. The elastic composite film is used as both a triboelectric layer and an electrode in the fully enclosed TENG structure. Furthermore, the sponge structure was highly flexible since the quick increase in stress in reaction to strain may be attributable to the collapse of pores in the densification area [118]. The sponges totally adjusted to their initial structure when subjected to modest strains. Even in the severe scenario of 80 percent distortion, the sponge′s shape return rate was much higher than 50 percent. The sponges′ toughness and flexibility are related to their distinctive interconnected 3D network with chemical crosslinking [119].

**Figure 9:**
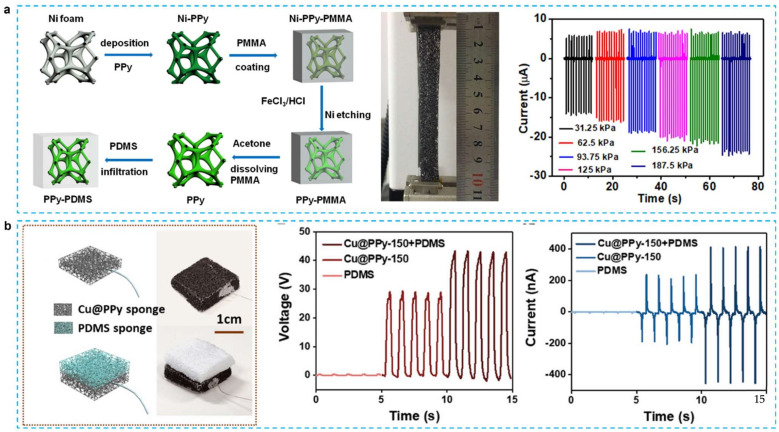
(**a**) Stretchable 3D polymer for simultaneously mechanical energy harvesting and biomimetic force sensing [29]. (**b**) Elastic Cu@PPy sponge for hybrid device with energy conversion and storage [27].

**Figure 10:**
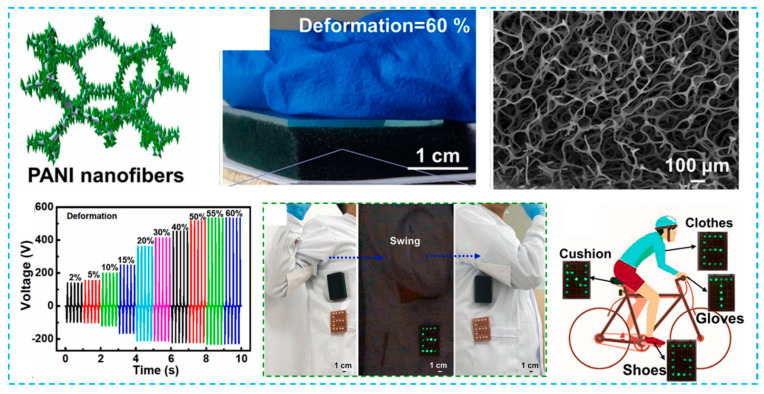
Conductive PANI fibers on PU elastic sponge-based TENG for effective random mechanical energy harvesting. The LEDs are lighted by the ES-TENG during swinging the arm, which liiustrate the ES-TENG for harvesting irregular and random energy on various flexible object surfaces [104].

The voltage output of a single-electrode TENG constructed by merging PDMS friction layer within 3D PPy network was three times greater than that of typical TENGs [29]. As shown in Figure 9a, the friction between the porous PPy and the porous PDMS in the TENG causes electron injection from the PPys to the PDMS. When the force is removed, the neighboring PPy electrodes produce a positive charge. They flow from PPy to ground until they reach equilibrium. Combining it with PDMS and porous PPy not only greatly enhances the electric output signals (11.3 mA/m^2^), but also makes it exceedingly flexible and stretchable (310% length increase). Figure 9b exhibited a copper sponge-based triboelectric nanogenerators, which has single electrode with varying the PPy content and hybridization of Cu@PPy with PDMS sponge to turn the voltage outputs [27]. The average voltage peak of 50 V, current of 400 nA, and charge of 20 nC were obtained, respectively. The practicality and accessibility of producing metal sponge-based elastic TENG were established by these findings. By using a simple high-temperature carbonization method, Zhao and coworkers were able to construct TENGs made of melamine foam [120]. This can help spread the organic electralyte into the electrons using the results of hydrophobial carbonized melamine foams, which include uniform and micrometer pores. It was also shown that pulsed energy was stored with CMF-based HF-SCs at frequencies ranging from 40 to 1350 Hz. The energy utilization efficiency of the CMF-based CFS was raised by 20.3 percent at 1350 Hz when compared to conventionally used carbon-based SCs (AC-SCs), showing that HF-SCs are more beneficial than conventional AC-SCs in storing pulse energy of variable frequencies. However, the above work did not show the energy storage application usage in relation to human acitvity. Very recently, Liu et al. further developed the TENG using the combination of PANI nanofibers with elastic sponge to act as electrode [104]. As illustrated in Figure 10, aniline was coated using a dilute chemical polymerization process on the surface of a sponge with a 3D reticular structure, which enables it to create around 60% elastic deformation. When compression deformation is increased from 2% to 60%, the voltage increases from 142 V to 520 V. For example, it can be seen that the electric energy generated by the ES-TENG can power the LED lights during the arm swing, suggesting that the ES-TENG can gather random-rotation, mechanical energy from the environment. Due to the flexibility of ES-TENG, it may be utilized on a variety of flexible object surfaces, including gloves, clothing, pillows, and shoes, to gather irregular and random mechanical energy.

Cellulose-based materials have been used in flexible energy harvesting and storage systems since of their low density, elasticity, ease of manufacture, large specific area, and superior mechanical characteristics [117,121]. Because of its high oxygen content, cellulose appears to have lost electrons and is efficiently positively charged, making it a promising positive material for polymer-based environmentally friendly TENGs [122], for example, cellulose nanofibrils, which are made from cellulose with high aspect ratios, outstanding mechanical qualities, great flexibility, and favorable electrical properties [123]. They are being increasingly investigated as energy storage and harvesting materials that are adaptable [124]. Because of its proclivity to shed electrons, cellulose has traditionally been regarded as a positive substance in the triboelectric family of materials [125]. Natural cellulose, on the other hand, has just a weak triobolary, which results in worse performance when utilized to manufacture TENGs because to its low polarity [126].

**Figure 11:**
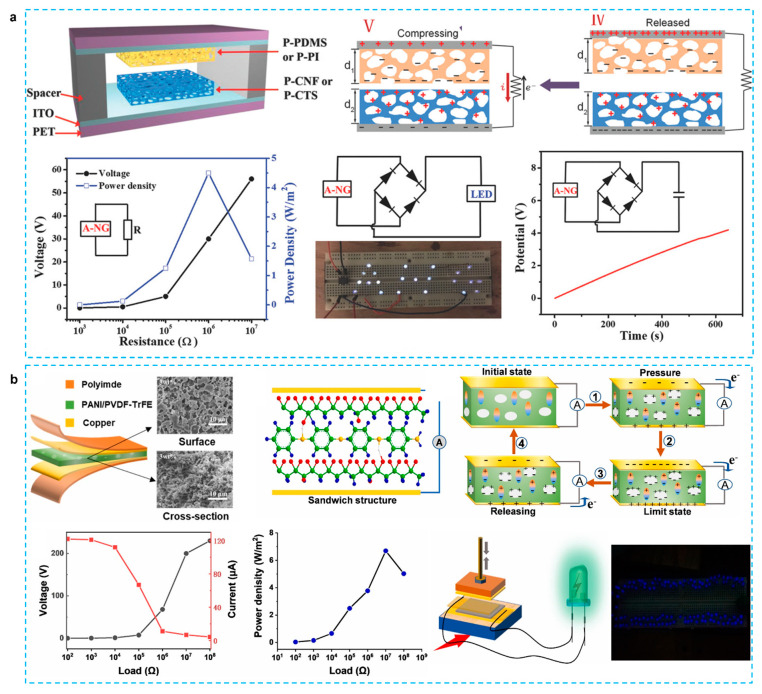
(**a**) Highly porous cellulose nanofibril and Chitosan aerogel based triboelectric nanogenerators [128]. (**b**) PANI/PVDF-TrFE porous aerogel bulk piezoelectric and triboelectric hybrid nanogenerator based on in-situ doping and liquid nitrogen quenching [60].

In contrast, although cellulose has demonstrated its good candidate as a positive friction material, it still be considerably weak for its triboelectric outputs due to the low surface charge density. Chemical modification, surface patterning, dipole orientation, and structure optimization have all been used to enhance the surface charge density of polymers [127]. As shown in Figure 11a, Zheng et al. first reported on porous polymer aerogel-based TENGs with aerogels as both positive and negative materials to enhance triboelectric performance [128]. They proposed a novel design to add Chitosan as a positive material for fabricating aerogel TENG, because amino groups in Chitosan have excellent electron-donating functionality to obtain larger tribopositive polarity contrasted to normal cellulose [129]. The device′s triboelectric outputs rise dramatically as porosity increases, due to an increase in contact area and electrostatic induction in the porous structure, resulting in increased charges on the porous surface. Using cellulose aerogel as a TENG material, a very high voltage of 60.6 V, a current of 7.7 A, and a power density of 2.33 W/m^2^ were achieved, bringing fresh insights into the investigation of porous materials with variable triboelectric polarities for high performance TENGs. Using PEDOT:PSS/graphene oxide composite aerogel sponges for TENG, the PEDOT:PSS network were bonded to the surface of the GO with abundant halogen functional groups [28]. The curving energy of the graphene surface is altered by these halogen functional groups and aromatic hydrocarbons. As a result, the holes and curves might be present indefinitely to form these sponges. Another novel work reported that cellulose aerogel-based TENG can also be used as a power source to electropolymerize conducting polymers on a carbon nanotube (CNT) electrode, thus saving energy and opening a new approach for electrochemical synthesis [130]. Ouyang et al. presented a portable TENG-based transdermal drug delivery system for precise and on-demand drug dosing [99]. The PPy is the drug supplier because it offers superior conductivity, stability, biocompatibility and easy modifications to the surface. The system also has achieved customizable drug release rates for transdermal pharmaceutical delivery. By altering the duration of TENG loads or the power control resistance, the rate can be set from 0.05 to 0.25 μg/cm^2^ per minute. Furthermore, the performance of such TENG-based medicines delivery systems is validated by ex vivo trials with a 50 percent improvement over standard transdermal patches. It should be emphasized that the proper preparation processes for porous materials, as well as the selection of fillers, are critical for the manufacture of outstanding nano-generator devices. However, compared to intensive study on 3D porous structured TENG, there are not many PENG devices using 3D porous structure. Very recently, as shown in Figure 11b, Yu et al. used PVDF-TrFE copolymer as the matrix to prepare composite aerogel via freeze-drying method and introduced PANI for improving conductivity and sodium carboxymethyl cellulose (SCMC) for increasing porosity [60]. The device produced 246 V and 122 A at 30 Hz and 0.31 MPa, has a power density of 6.69 W/m^2^, and can directly light up 119 blue LEDs in succession.

### 3.3. Textile Based TENGs

Similar to PENG, TENGs are a potential mechanical energy-harvesting power supply based on textiles, and there has been an increasing attempt to combine TENGs with fabrics. The incorporation of the TENG without affecting its function or the original features of the textile, such as look, breathability, washability, durability, and feel, is a significant challenge [131]. However, large-scale production of the textile-based and self-powered sensor is still a challenge. First, a weaving operation is required for the fabric-based TENG, which adds to the production time and expense. Second, fabric-based TENGs have low stretchability, complex manufacturing, and poor wearing comfort. Third, modern textile-based TENGs were designed for a single-usage scenario involving a single or few body components. Wearable sensors should be able to respond to mechanical stimuli from diverse body locations, such as pressing, bending, and twisting, stretching.

**Figure 12:**
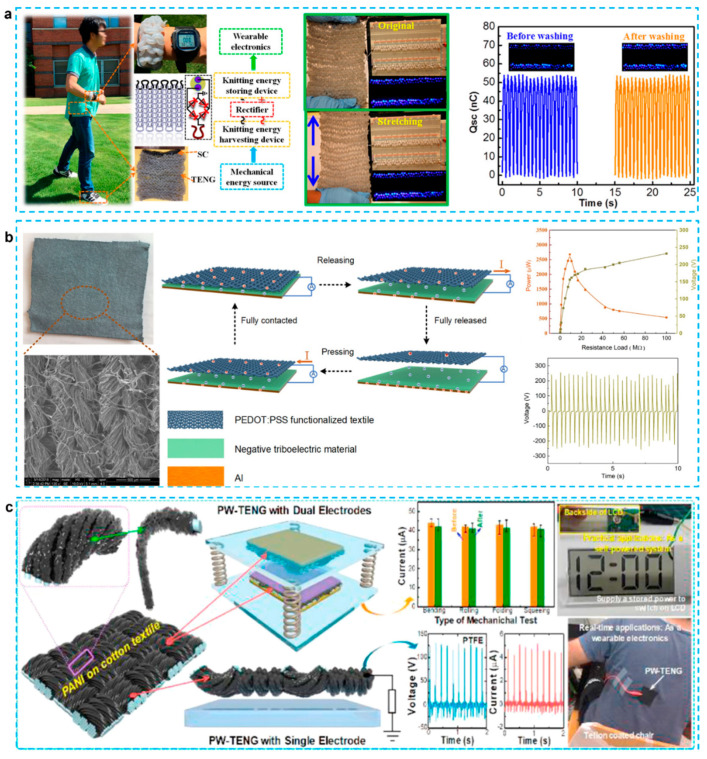
(**a**) A highly stretchable and washable all-yarn-based self-charging knitting power textile composed of fiber triboelectric nanogenerators [105]. (**b**) Multi-functional and PEDOT:PSS based TENG was made from the stretchable smart textiles to harvest energy from various body motions [132]. (**c**) Wearable and durable triboelectric nanogenerators via polyaniline coated cotton textiles as a movement sensor and self-powered system [33].

The all-yarn-based, self-charging textile, using CNF/PEDOT:PSS electrodes has been proposed for sustainably powering wearable electronics. The device used a highly soft, flexible, and stretchable material, and obtained the maximum peak power density of ∼85 mW·m^−2^ [105]. As depicted in Figure 12a, before or after stretching, TENG fabric can flash 124 LEDs in a row. TENG yarn-based fabrics have strong electrical output performance and flexibility due to their knitting structure. The TENG fabric is lighter, waterproof, and anticorrosive, more than earlier wearable power sources based on solid elements such as metal. The TENG fabric can still light LEDs after being immersed in water and dried naturally, indicating that our TENG fabric can be washed even after being damaged. In another work illustrated in Figure 12b, to create the PEDOT:PSS functionalized textiles, a simple and low-cost dip coating technique is described [132]. This simple and low-cost technology is easily adaptable to large-scale manufacturing of functionalized fabrics. With a layer of PEDOT: PSS coated textile and PTFE under foot stepping, a large output density of 2 W/m^2^ is obtained at 2 Hz, and the matched impedance is as low as 14 MΩ. Besides the PEDOT, PANI was also reported in a textile TENG demonstrating a low-processing cost with superior electrical performance and durability [33]. As shown in Figure 12c, to construct a TENG, a PANI coated worn-out cotton textile (PANI@WCT) is used as a positive triboelectric material and electrode. Both vertical contact-separation dual-electrode mode and single-electrode mode TENG using PANI on worn-out cotton textile were studied. The PANI@WCT developed at a 20-h deposition period is an ideal sample for achieving high output performance. PANI@WCT-based TENG′s electrical stability and mechanical endurance.

## 4. Conclusions and Outlook

In conclusion, high-performance, stretchable and uniform nanogenerators are excellent options for human energy conversion. In the past years, due to the huge advancement and development of nanostructured materials, especially conducting polymers, PENG and TENG have been intensively investigated. In this review, the CPs-assisted PENG and TENG with different configurations are discussed and illustrated. The ability of CPs to be solution processed and polymerized in situ contributes to the ease of fabricating their composites in a variety of wearable device forms, such as fibers, nanorods, films, sponges, foams, aerogels, and textiles. The use of composites of conductive polymers to overcome brittleness and processability while keeping electrical conductivity and desirable biological features such as cell adhesion has been investigated. The required electrical conductivity of conductive polymers is routinely sacrificed in order to improve the mechanical characteristics of electrodes. A fundamental understanding of the relation between both the conductive polymer filler and the non-conductive polymer matrix would also result in a synergistic effect in the mechanical and electrical properties of the composites. For minor strain detection and energy harvesting, conformal and nanostructured CP helps boost a stable signal between the electrode and soft skin. However, there are still significant challenges to solve in the practical application of conducting polymer-based nanogenerators, as detailed below.

First, it is important to improve the performance of CPs themselves. Controlling the electrical conductivity of pure CPs is difficult. Future study should focus on novel synthetic methodologies and assembly technologies for mass-producing CPs nanoparticles. CPs with great structural order are known to be electrically conductive. It is essential to propose effective strategies for managing the crystalline/amorphous ratio in CP structures. This will be an important study area for CP-based nanomaterial fabrication techniques. Choosing the right CPs and preparing them correctly appears to be the key to successful nanocomposites. This field could benefit greatly from future research on CPs with acceptable sizes, nanostructure, and properties. Simple, efficient, scalable, and economical nanomanufacturing of multi-component nanocomposites is required.

Second is to conduct large-scale, green-fabrication methodology. Despite major advancements in flexible nanogenerator technology, there is still much work to be done in terms of device integration and devising large-area, low-cost, clean-room, pollutant-free procedures. There are two parts to this issue that stem from device production and long-term usage: the chemical compatibility and environmental stability of the device, and the degradation of the nanogenerator to reduce e-waste. The key research path in the future will be to produce textile-based nanogenerators with stable and machine-washable performance. It is preferable that all of the materials used are widely acknowledged by the textile industry and that the device is constructed utilizing miniature industrial machinery.

Third is to achieve all-in-one integrated electronics with standardized evaluation. Development of multifunctional sensing and energy-harvesting systems that may be used for continuous monitoring and power support of patient′s health status is urgently required. Because of its versatility, an all-in-one electronics system that includes strain/pressure sensors, temperature sensors, a gas sensing device for measuring humidity, electrophysiological (EP) sensors, and energy harvesting devices with wireless signal communication is highly desirable for both daily and clinical applications, as previously stated. In addition, it is crucial for standardized evaluation and application of nanogenerator technologies on various configurations.

## Figures and Tables

**Table 1:** Comparison of performance of selected conducting polymer-based PENG.

Contact Material		Electrode	Structure	Output Performance				Durability (Cycles)	Ref.
Filler	Matrix			Voltage	Current	Load Resistance	Power Density		
PEDOT-C4:DS	PEDOT:PSS	AgNW, PEDOT:PSS	Sandwich	1.54 V	166.0 nA	9 MΩ	63.0 nW	25,000	[59]
PANI	PVDF-TrFE	Copper foil	Sandwich	246 V	122 μA	100 Ω–100 MΩ	$6.69\;W/m^{2}$	-	[60]
PVDF	Cu film	PANI	-	-	55.9 nA	-	-	-	[22]
PVDF, BaTiO3, MoS2	PMF NMP	AgNWs/PEDOT: PSS	Sandwich	750 mV	-	-	-	5000	[61]
PEDOT	PVDF NFs	PVDF NF	3D Multilayer	48 V	6 μA	30 MΩ	51 μW	21,000	[62]
PANI/g-C3N4	PVDF	copper	PVDF/ PANI/g-C3N4/ PPBF	~30 V	3.7 μA	8 MΩ	14.7 μ $W/{\mathrm{cm}}^{2}$	50,000	[63]
CsPbBr3	PVDF NFM	PPy	3D multilayer	10.3 V	1.29 μA $/cm^{2}$	20 MΩ	3.31 μW	-	[30]
SCP/ZnO QDS	PVDF	PEDOT:PSS.Ag	3D multilayer	1.46 V	4.82 μA $/cm^{2}$	-	0.97 m$W/{\mathrm{cm}}^{3}$	3500	[64]
PANI/ZnS	P(VDF-HFP)	Carbon tape	coreshell	3 V	-	-	2.92 μ$W/{\mathrm{cm}}^{2}$	-	[44]
PANI nanochain	PVDF	Ag	-	4.2 V	0.85 μA $/cm^{2}$	-	3.56 μ $W/{\mathrm{mm}}^{3}$	-	[65]
CNT	PVDF	PEDOT:PSS	-	1.2 V	3.8 nA	~9 Ω	-	-	[66]
Ga/ZnO	PEN	AI/PEDOT:PSS	-	398 mV	8.4 μA	9 MΩ	0.78 μ $W/{\mathrm{cm}}^{2}$	-	[45]
HBA CEA	PEDOT:PSS	PEDOT:P(SS-co-HBA)PEDOT:P(SS-co-CEA)	Sandwich	4.12 V	817.3 nA	-	847.5 nW	1000	[67]
HNT PANI	PVDF	PVDF	Sandwich	7.2 V	0.75 μA	0.5~15 MΩ	0.25 μ $W/{\mathrm{cm}}^{2}$	2000	[68]
PCBM61	PVDF	Ag/Ag with MoO3	3D multilayer	43.1 V	589 nA	-	-	-	[69]
-	PVDF	AgNWs/PEDOT-C6:DS	3D multilayer	7.02 V	1.11 A	1–11 MΩ	1.18 W	20,000	[26]
TCA	PEDOT:PSS	TCA	nanorods	0.72 V	-	~13.9 KΩ	0.25 μW	-	[46]

**Table 2:** Comparison of performance of selected conducting polymer-based TENG.

Contact Material		Electrode	Structure	Output Performance				Durability (Cycles)	Ref.
Positive	Negative			Voltage	Current	Load Resistance	Power Density		
PPy	PS	PTFE/Cu	Radial arrayed	1.05 V	-	1 MΩ–3.75 MΩ	-	-	[99]
PANI	PVDF	N-PANI	Arch-shaped	1186 V	45.70 μA	100 MΩ	87.1 μ $C/{\mathrm{cm}}^{2}$	15,000	[100]
PEDOT:PSS	ITO	AgNW	bilayer	160 V	>50 μA	2 MΩ	$>15\;W/m^{2}$	-	[101]
PANI@WCT	-	PANI@WCT	cotton textile	460 V	55 μA	7.8 ± 2.1 kΩ	$11.25\;W/m^{2}$	5000	[33]
The snow	PEDOT:PSS	AI	micropatterned Si layer	8 V	40 μA $/m^{2}$	50 MΩ	$0.2\;\mathrm{mW}/{\mathrm{cm}}^{2}$	8000	[102]
PAM	PEDOT:PSS	MGP hydrogel	hydrogel and sandwich	383.8 V	26.9 μA	30 MΩ	$1250\;\mathrm{mW}/m^{2}$	16,000	[103]
PANI NW	elastic sponge	sponge	3D reticular structure	540 V	6 μA	20 MΩ	280 μW	30,000	[104]
PEDOT:PSS	CNF	CF	3D nanonetwork	150 V	2.9 μA	100 MΩ	$85\;\mathrm{mW}/m^{2}$	6000	[105]
Human skin	MT-PDMS	PPy@CT	-	200 V	6.5 μA	70 MΩ	82 μ $W/m^{2}$	5000	[106]
PTFE	hPPy	PPy	Sandwich-structure	48 V	$30\;\mathrm{mA}/m^{2}$	100 MΩ–1 GΩ	$5.5\;W/m^{2}$	10,000	[97]
PDMS	hogskin	PEDOT:PSS	-	255.6 V	22.6 μA	100 MΩ	$4.06\;\mathrm{mW}/m^{2}$	200	[107]
Human skin	Silicone rubber	PEDOT:PSS	Sandwich with liquid	265 V	24.9 μA	100 KΩ–10 GΩ	24.8 μW	1000	[108]
